# Spotlight on Plant Bromodomain Proteins

**DOI:** 10.3390/biology12081076

**Published:** 2023-08-02

**Authors:** Eirini Bardani, Paraskevi Kallemi, Martha Tselika, Konstantina Katsarou, Kriton Kalantidis

**Affiliations:** 1 Department of Biology, University of Crete, Voutes University Campus, 71500 Heraklion, Greece; eirini_bardani@imbb.forth.gr (E.B.); pkal9.pk@gmail.com (P.K.); mar8a01_@hotmail.com (M.T.); 2 Institute of Molecular Biology and Biotechnology, Foundation for Research and Technology-Hellas, 70013 Heraklion, Greece

**Keywords:** acetylation, acetyltransferase, histone, chromatin-remodeling, SWI/SNF, BET, SANT

## Abstract

**Simple Summary:**

Bromodomains are protein modules able to read the chromatin epigenetic state and translate it into gene expression. Extensive research has been conducted on bromodomain-containing proteins in humans, particularly in the context of disease and cancer. However, in plants their functions remain far less understood. The focus of this review is to comprehensively organize plant bromodomain proteins into functional groups and elucidate what is currently known about their functions, with an emphasis on the model plant Arabidopsis. By systemically examining these proteins, we aim to shed light on their diverse roles, highlight members with yet undetermined functions, present similarities and differences with other eukaryotic homologs and pinpoint unresolved questions that present exciting opportunities for future research in this field. We anticipate that this review will contribute to a deeper understanding and inspire further investigations into the functions of these remarkable proteins in plants.

**Abstract:**

Bromodomain-containing proteins (BRD-proteins) are the “readers” of histone lysine acetylation, translating chromatin state into gene expression. They act alone or as components of larger complexes and exhibit diverse functions to regulate gene expression; they participate in chromatin remodeling complexes, mediate histone modifications, serve as scaffolds to recruit transcriptional regulators or act themselves as transcriptional co-activators or repressors. Human BRD-proteins have been extensively studied and have gained interest as potential drug targets for various diseases, whereas in plants, this group of proteins is still not well investigated. In this review, we aimed to concentrate scientific knowledge on these chromatin “readers” with a focus on Arabidopsis. We organized plant BRD-proteins into groups based on their functions and domain architecture and summarized the published work regarding their interactions, activity and diverse functions. Overall, it seems that plant BRD-proteins are indispensable components and fine-tuners of the complex network plants have built to regulate development, flowering, hormone signaling and response to various biotic or abiotic stresses. This work will facilitate the understanding of their roles in plants and highlight BRD-proteins with yet undiscovered functions.

## 1. Introduction

Proteins, once produced, can be modified by a vast number of post-translational modifications (PTMs) [1], which can affect protein interactions, enzyme activities or other functions. Acetylation is one of the most prominent PTMs, together with phosphorylation and ubiquitination [2]. Acetylation takes place at the α-amino group of the N-terminus of proteins or at the ε-amino group of internal lysine residues. Both are omnipresent in living organisms and are performed by acetyltransferases, which transfer acetyl groups from Acetyl-CoA to their substrates [3]. In addition to enzymatic acetylation, non-enzymatic acetylation has also been described both in eukaryotes and prokaryotes, driven by the concentrations of acetyl-CoA and acetyl-phosphate, respectively [4]. In higher eukaryotes, the majority of cytosolic proteins are acetylated at their N-terminus, while specific proteins, mainly histones, are acetylated at internal lysine residues [5]. Histone acetylation has been associated with “open chromatin” and gene activity. As lysine acetylation neutralizes the charge of the ε-amino group, it reduces the interactions between histones and DNA, affecting both nucleosome structure and chromatin organization [6]. Studies in yeast and Drosophila have highlighted the importance of the specific histone acetylation for the regulation of chromatin state and gene activity [7,8]. The “writers” and “erasers” of acetylation were soon identified as histone acetyltransferases (HATs) and histone deacetylases (HDACs), respectively [9,10]. Domains acting as acetyl-lysine “readers” have also been discovered, with the most prominent being the bromodomains (BRDs), first discovered in the brahma gene of Drosophila [11]. It is to note that, besides bromodomains, additional domains such as YEATS (Yaf9, ENL, AF9, Taf14, Sas5) and DPF (double plant homeodomain finger) domains are emerging as readers of histone lysine acetylation [12,13].

BRDs usually coexist in proteins with other interaction or catalytic domains, including methyl-readers such as plant homeodomain (PHD) fingers, histone acetyltransferase catalytic domains as well as multiple BRDs [14,15]. The ~110 amino acid BRD has a distinct architecture, forming four α-helices (αZ, αA, αB and αC) linked by two loops (ZA and BC), which construct a conserved hydrophobic pocket that recognizes acetylated lysine residues (Kac) in histones. A conserved asparagine (Asn) residue at the N-terminus of the BC loop is crucial for Kac recognition [16,17,18,19].

BRD-containing proteins perform diverse functions, acting alone or as parts of larger complexes and are involved in transcriptional regulation of gene expression. They can function in diverse ways: First, they can be components of chromatin-remodeling complexes (CRCs) that target chromatin and control its compaction and decompaction [14,20]. An example is the switch/sucrose non-fermenting (SWI/SNF) complex, carrying a core subunit that performs ATP-dependent alteration of chromatin structure, leading to open chromatin and subsequent accessibility to transcription factors [21,22]. Second, BRD-containing proteins can participate in histone modifications, directly catalyzing the deposit of PTMs on histones, such as HATs. Third, some BRD-containing proteins seem to have important roles as scaffolds that regulate the recruitment of transcriptional regulators to particular loci through recognition of acetylated histones. The Bromodomain-and-extra-terminal-domain (BET) family proteins, thoroughly studied in humans, attach to acetylated chromatin through their BRDs and recruit transcriptional regulators via their ET domain [23]. Finally, some BRD-containing proteins can function as transcriptional co-regulators, acting either as activators or repressors of transcription [14].

The number of BRD-proteins varies in different organisms. Human BRD members are 46 in total, are divided into eight distinct groups [15] and have been extensively studied as drug targets for diverse diseases [24,25,26,27]. Plants also have BRD-proteins, with their numbers varying from species to species: 29 BRDs have been identified in Arabidopsis [28], 22 in rice [29], 57 in *Glycine max* [28] and only 9 in nanoalga *Cyanidioschyzon merolae* [28]. Interestingly, in contrast to their human counterparts, the vast majority of plant BRD-proteins contain a single BRD, with only a few cases of multiple BRDs [28]. However, despite their abundance and diversity in plants, their functional roles are less explored. In this review, we focus on the different groups of BRD-containing proteins in plants, their domain architecture, as well as their interactors, activities and physiological roles described. We focus on Arabidopsis BRD-proteins, as the information on their domain architecture, phylogeny and functional roles is relatively more abundant.

## 2. Domain Architecture of Bromodomain-Containing Proteins in Arabidopsis

**Figure 1:**
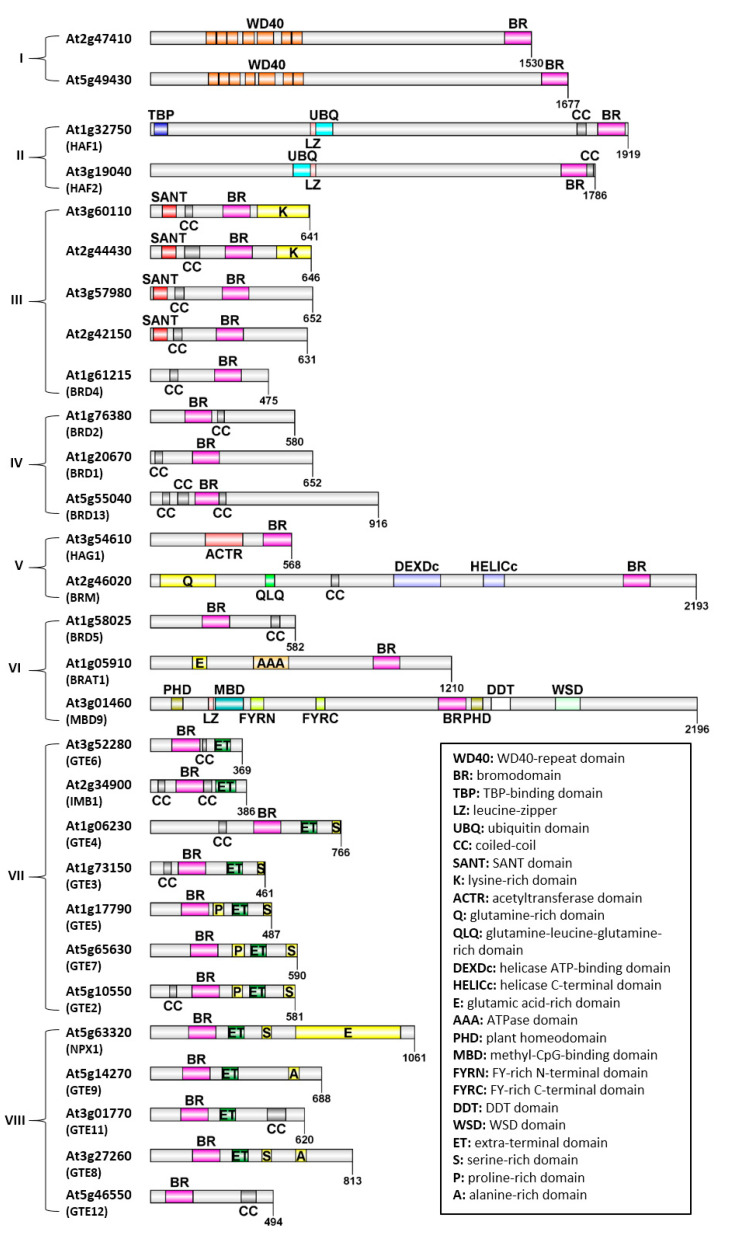
Domain architecture of Arabidopsis BRD-proteins. The relative length and conserved regions are depicted for each protein. Different domains are presented in different colors, and their names are explained at the bottom right corner. Phylogenetic clusters (adapted from [28]) are shown in brackets (I–VIII).

In Arabidopsis, 29 BRD-containing proteins have been identified, which belong to different families and contain a collection of additional domains [28,29]. In Figure 1, the relative protein length and the presence of different domains are highlighted. Arabidopsis BRD-proteins vary in length, with the smallest carrying 369 and the largest 2196 aminoacid residues (aa). Based on their domain architecture, the 29 proteins can be divided into subgroups, with the most prominent being a group of proteins carrying an ET domain adjacent to the bromodomain. Apart from the ET domain, various domains can be found in the rest BRD-proteins, such as a SANT domain, WD40-repeats, domains with HAT activity, as well as domains with ATPase activity. Additionally, several of the Arabidopsis BRD-proteins contain coiled-coils, which are involved in protein–protein interactions, and regions rich in specific aminoacids, such as lysine-rich, proline-rich, serine-rich and glutamine-rich domains. According to Rao and co-authors [28], Arabidopsis BRD-proteins can be divided in 8 distinct phylogenetic clusters (Figure 1):

(I) 2 proteins carrying WD40 repeats (At2g47410, At5g49430),
(II) 2 proteins with ubiquitin (UBQ) domain and HAT activity (HAF1/At1g32750 and HAF2/At3g19040),
(III) 4 proteins carrying an N-terminal SANT domain (At3g60110, At2g44430, At3g57980, At2g42150) and 1 protein with a single BRD (BRD4/At1g61215),
(IV) 3 proteins carrying a single BRD (BRD2/At1g76380, BRD1/At1g20670, BRD13/At5g55040),
(V) a HAT (HAG1/At3g54610) and a large multidomain ATPase (BRM/At2g46020),
(VI) a heterogeneous group of 3 proteins; 1 with a single BRD (BRD5/At1g58025), 1 carrying an AAA domain (BRAT1/At1g05910) and 1 carrying a methyl-CpG-binding domain (MBD9/At3g01460),
(VII) a group of 7 BET proteins (At3g52280/GTE6, At2g34900/GTE1, At1g06230/GTE4, At1g73150/GTE3, At1g17790/GTE5, At5g65630/GTE7 and At5g10550/GTE2); this group is mentioned as the first cluster of BET proteins in Section 4.1,
(VIII) the rest of the 5 BET proteins (At5g63320/GTE10, At5g14270/GTE9, At3g01770/GTE11, At3g27260/GTE8, At5g46550/GTE12); this group is mentioned as the second cluster of BET proteins in Section 4.2.

**Figure 2:**
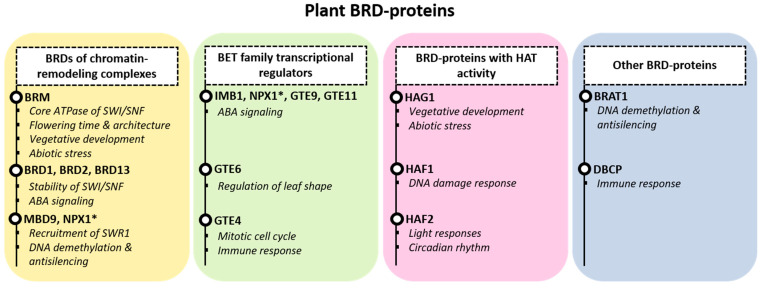
BRD-protein functions in plants. Plant BRD-proteins are divided into 4 groups. Members of chromatin-remodeling complexes (CRCs): BRM together with BRD1,2 and 13 are linked to the SWI/SNF CRC; MBD9 and NPX1 are linked to the SWR1 CRC (yellow box). BET family transcriptional regulators with known functions – IMB1, NPX1, GTE9, GTE11, GTE6, GTE4 – are shown (green box). Histone acetyltransferases of the GNAT (HAG1) and TAFII250 family (HAF1, HAF2) are shown in the pink box, and additional members (BRAT1, DBCP) are shown separately (grey box). For every member, the known function in brief is indicated. NPX1 is marked with an asterisk (*), as it is included in two groups.

From the BRD-proteins found in plant species, several have been characterized, but for many of them, their physiological role still remains elusive. In the next paragraphs, we will discuss the functions that have been described to date mainly for Arabidopsis BRD-proteins, regarding their interactions, activity and physiological roles. As their domain architecture is not completely aligned with their phylogeny, we divided them into four groups, primarily based on their function: (a) BRD-proteins of chromatin-remodeling complexes, (b) BET family transcriptional regulators, (c) BRD-proteins with histone acetyltransferase activity and (d) other BRD-proteins (Figure 2).

## 3. Bromodomain Proteins of Chromatin-Remodeling Complexes

### 3.1. BRD-Proteins of the SWI/SNF Complex

ATP-dependent chromatin remodeling complexes (CRCs) destabilize the interaction between histones and DNA to facilitate the accessibility of regulatory proteins to their target DNA sequences. The most studied class is Switch/Sucrose non-fermenting (SWI/SNF) and its catalytic ATPase subunit, named BRAHMA (BRM), first identified in yeast and later in Drosophila [30]. The SWI/SNF complex mediates the sliding of nucleosomes as well as the disruption of histone-DNA interactions, and these activities can be performed in vitro by the core complex, including the SWI/SNF2 ATPase—one SNF5 subunit and two copies of SANT/SWIRM/Leu zipper (SWI3) subunits [31,32]. In Arabidopsis, there are four SNF2-ATPases—BRAHMA (BRM), SPLAYED (SYD), MINUSCULE 1 (MINU1) and MINUSCULE 2 (MINU2), from which only BRM carries a bromodomain. BRM (At2g46020) is a large protein of 2193aa, containing an N-terminal glutamine (Q)-rich region, a glutamine-leucine-glutamine (QLQ)-rich region, as well as a central catalytic helicase-like ATPase domain formed by the DEXDc and the HELICc domain. The BRD is found in the C-terminal region of this protein and recognizes acetylated lysine residues in histones H3 and H4 (Figure 1) [33,34].

BRM is involved in numerous and diverse processes in plants and has been recently reviewed [35]. Several studies have highlighted the role of BRM in embryo development and embryogenesis [36,37]. Later in development, BRM negatively regulates vegetative phase genes by directly fine-tuning the expression of *mir156* [38]. An involvement of BRM in the regulation of photomorphogenesis through direct interaction with PHYTOCHROME-INTERACTING FACTOR 1 (PIF1) has also been reported [39]. Furthermore, BRM is implicated in cell proliferation processes regulated by the SWI/SNF-ANGUSTIFOLIA3 module, which acts as a major player at the transition from cell proliferation to cell differentiation in a developing leaf [40]. In roots, BRM regulates stem cell niche maintenance through the auxin distribution pathway and directly targets several PIN-FORMED (PIN) genes [41]. BRM is involved in the regulation of the cytokinin (CK) pathway, as *brm* mutants exhibit inhibition of growth after CK application and more pronounced phenotypic alterations, such as leaf serrations and increased leaf width [42]. BRM and TCP factors (TEOSINTE BRANCHED 1, CYCLOIDEA, PROLIFERATING CELL FACTOR1/2) regulate CK responses to promote leaf growth. BRM interacts with TCP4 both in vivo and in vitro, inducing the expression of ARABIDOPSIS RESPONSE REGULATOR 16 (ARR16), an inhibitor of CK response [42].

Another important role of BRM is the regulation of flowering time. *brm* mutants flower earlier in long days, suggesting that BRM represses flowering [43]. In short days, *brm* mutants flower late, with a number of plants not flowering at all. Further analyses indicate that BRM represses the flowering genes CONSTANS (CO), FLOWERING LOCUS T (FLT), SUPPRESSOR OVEREXRPESSION OF CO1 (SOC1) and FLOWERING LOCUS C (FLC) and activates flowering repressor SVP (SHORT VEGETATIVE PHASE) [44]. Recently, BRM was found to participate in Gibberillin (GA) signaling-mediated flowering through the formation of a DELLA-BRM-NUCLEAR FACTOR YC (NF-YC) module in Arabidopsis, which impairs the binding of NF-YC to SOC1, leading to late flowering [45]. However, when GA is present, DELLA degradation disrupts the DELLA-BRM-NF-YC module and, as a result, SOC1 is upregulated, leading to early flowering [45]. This finding further explains the role of BRM in the control of flowering time.

In addition to flowering time, BRM activates floral homeotic genes and affects floral architecture [33]. *brm* mutant flowers present several developmental abnormalities due to the role of BRM in the activation of transcriptional activators LEAFY (LFY) and SEPALLATA3 (SEP3), as well as BRM interaction with BREVIPEDICELLUS (BP), a key regulator in inflorescence architecture [34,46,47].

BRM regulates responses to abiotic stress, including functions related to abscisic acid (ABA). Repressed ABA responses are related to the growth defects of *brm* mutants, probably by direct repression of ABA INSENSITIVE5 (ABI5) [48]. Type 2C protein phosphatases (PP2Cs) and sucrose non-fermenting 1-related protein kinases (SnRK2s)—key components of the ABA pathway—physically interact with BRM, changing its phosphorylation status [49]. In *brm* mutants, PP2C expression is induced under salt stress, suggesting a role for BRM in the alterations of PP2C chromatin status [50]. In the context of abiotic stress, BRM was also found to participate in phosphate starvation response, as *brm* mutants showed hypersensitivity to phosphate-induced inhibition of root growth. BRM represses LOW PHOSPHATE ROOT1 and 2 (LPR1-LPR2) expression by decreasing H3 histone acetylation levels, most likely by recruiting HISTONE DEACETYLASE 6 (HDA6) to these loci [44]. Regarding TOR (TARGET OF RAPAMYCIN)-mediated stress responses, when stress inhibits TOR, BRM releases the TOR-repressed stress-specific genes [51]. BRM also interacts with MMS21 (METHYL METHANE SULFONATE SENSITIVITY 21), which is involved in DNA damage [52] and stress response [53]. Moreover, Sakamoto and co-authors demonstrated that BRM degradation promotes boron tolerance in Arabidopsis, as it prevents susceptibility to DNA damage caused by histone hyperacetylation [54]. Another aspect of BRM is its participation in heat-shock stress, together with FORGETTER1 (FGT1); *brm* mutants have depleted heat-shock memory, and it has been proposed that the interaction between BRM and FGT1 mediates stress-induced chromatin memory by preventing nucleosome recovery at these loci [55].

Interestingly, Wang et al. [56] demonstrated a different role of BRM as a partner of the Microprocessor component Serrate (SE). BRM can bind and unwind pri-miRNAs, inhibiting their processing. Additionally, the secondary structures of pri-miRNAs differ between *brm* mutants and *wild-type* (*wt*) plants. BRM interacts with pri-miRNAs through SE and remodels their secondary structure, preventing downstream processing by DCL1 and HYL1 [56].

Recently, another family of BRD-proteins has been identified as members of the SWI/SNF complex in Arabidopsis, further supporting the hypothesis that multiple BRD-proteins function as homologs of polybromoproteins found in animals. Specifically, BRD1 (At1g20670), BRD2 (At1g76380) and BRD13 (At5g55040) were co-purified with subunits of Arabidopsis SWI/SNF complexes [57]. Subsequent studies have demonstrated that BRD1, BRD2 and BRD13 are unique components of Arabidopsis SWI/SNF complexes, thereby introducing the presence of non-ATPase BRD-containing subunits in these complexes [58,59,60]; however, note that they do not contribute equally to their formation of BRM-containing SWI/SNF complexes [60]. Comparative analysis indicated that BRD1, BRD2 and BRD13 are highly similar and clustered into a single phylogenetic cluster. BRD1 and BRD2 are 60% identical at the amino acid level, while BRD13, which contains a longer C-terminal region, is 45% and 41% identical to BRD1 and BRD2, respectively. Apart from the conserved BRD, they contain one or more coiled-coil domains responsible for protein–protein interactions (Figure 1) [58]. Interestingly, these BRD-proteins exhibit a higher degree of similarity to the human SWI/SNF subunits BRD7 and BRD9 than to other BRD-proteins found in Arabidopsis [58].

*brd1, brd2* and *brd13* mutants display early flowering compared to *wt* plants, indicating that BRD1, BRD2 and BRD13 act as repressors of flowering. Since double mutants presented pronounced phenotypes, a functional redundancy of these BRD-proteins is thought to occur, even though the levels of redundancy differ between them [58,59,60]. In comparison to single and double mutants, *brdx3* triple mutants exhibited a general reduction in size, along with the production of smaller, curled leaves, shorter roots, early flowering and reduced fertility [58,59], a phenotype similar to *brm* mutants [58,59]. Quadruple *brm brdx3* mutants had no extra phenotypes and were very similar to *brm* mutants. These data support the idea that BRD1, BRD2 and BRD13 act as bona fide components of Arabidopsis SWI/SNF complexes [58] and that they act together with BRM to direct plant development [59]. Moreover, ABA- and GA-related phenotypes and the misexpression of ABA- and GA-responsive genes in *brdx3* and *brm* mutants demonstrate that BRD1, 2 and 13 are critical for SWI/SNF-mediated regulation of ABA and GA responses [58].

The bromodomains of BRD1, BRD2 and BRD13 bind H4K5ac and H4K8ac in vitro, and their accumulation around these acetylated histone-binding sites suggests their preferential association [59]. However, in *brdx3* mutant, the levels of H4K5ac and H4K8ac were not significantly reduced, indicating that BRD1, 2 and 13 are not directly involved in histone H4 acetylation deposition in the Arabidopsis genome [59]. Nonetheless, their bromodomains recruit BRD-containing SWI/SNF complexes to chromatin, and depletion of BRD1, 2 and 13 leads to decreased BRM protein levels. Thus, these BRD-proteins are essential for both SWI/SNF complex recruitment and maintaining the physiological abundance of BRM proteins in plants, which is crucial for the interaction of BRM with chromatin [59].

### 3.2. Bromodomain Proteins of the SWR1 Complex

The chromatin-remodeling complex SWR1 is associated with biotic and abiotic stress responses and is also important for flowering time, flower architecture, hypocotyl elongation and regulation of root growth [61]. SWR1 role is the deposit of H2A.Z, a conserved histone variant linked to active transcription, directly to nucleosomes. [62]. Arabidopsis share one or more orthologs with conserved subunits of yeast and human SWR1 complexes [63]. Additional components, such as MBD9 (METHYL-CPG-BINDING DOMAIN 9), CHR11/17 (CHROMATIN-REMODELING FACTOR 11/17) and TRA1a/1b (TRANSCRIPTION-ASSOCIATED PROTEIN 1A AND 1B), have been detected exclusively in plant SWR1 complexes [64]. MBD9 is a member of the methyl-CpG-binding family containing six domains: an MBD domain, a BRD, two PHD domains, FYRN and FYRC regions, a DDT domain as well as a WSD domain [64] (Figure 1). Notably, the MBD domain is unable to bind to methylated or unmethylated DNA due to the absence of crucial conserved residues necessary for methyl-CpG binding [63,64]. The MBD domain is not conserved among angiosperms, making the presumed capability of MBD9 to bind methylated DNA unlikely to be necessary for its function in plants, while DDT, BRD and the second PHD are quite conserved [64].

Arabidopsis *mbd9* mutants have pleiotropic phenotypes, including early flowering, attributed to the repression of FLC and multiple lateral branches [65]. MBD9 is found to preferentially localize in nucleosome-depleted regions, directly upstream of H2A.Z nucleosomes, driving SWR1 to localize at specific genes [66]. MBD9 is not a core subunit of the SWR1 Arabidopsis complex and is unable to influence open chromatin itself, with a possibility that is a component of a limited subset of SWR1 complexes in Arabidopsis [63]. Furthermore, it has been demonstrated that MBD9 functions as a bridge protein among known SWR1 components and ISWI (Imitation Switch) chromatin-remodeling regulators such as CHR11/17, suggesting a distinct mechanism of chromatin regulation [64]. Additionally, MBD9 interacts with the ISWI family of CRCs, revealing extra roles at nucleosome remodeling outside of H2A.Z deposition [66].

Another BRD-protein was found to cooperate with MBD9 and participate in the SWR1 complex. This protein is called NUCLEAR PROTEIN X1 (NPX1) and is a member of the BET family transcriptional regulators that will be discussed in detail in Section 4.2. MBD9 and NPX1 recognize specific chromatin marks generated by increased DNA methylation complex and guide SWR1 to deposit H2A.Z. It has been proposed that this deposition recruits REPRESSOR OF SILENCING1 (ROS1) to prevent DNA methylation and gene silencing [67].

## 4. BET Family Transcriptional Regulators

BET (Bromodomain and Extra-Terminal domain) proteins in plants are characterized by the simultaneous presence of one or two BRDs and an extra terminal domain (ET) (Figure 1). The ET domain is a protein–protein interaction motif that functions as part of non-canonical serine kinase [68,69]. The ET domain can be separated into three regions: the NET (N-terminal ET) domain, an intermediate sequence and the C-terminal SEED motif. From these, only the NET domain is conserved in all BET proteins. The conservation of the BRD and NET domain suggests that there are common functions in BET proteins and that within species there must be at least partial functional redundancy [20]. In contrast to animals and yeast [25], plant BET proteins contain only one BRD instead of two, and there are indications that the plant BRD exhibits more similarities with the second BRD of animal and yeast BET proteins [20]. It has been proposed that plant BET proteins form homodimers to overcome the lack of two BRDs, which takes place via a plant-specific amphipathic domain [20,70,71]. Most metazoan BET homologs interact with other transcription factors and mediate critical functions including cell proliferation, transcriptional regulation and innate immune response [72,73]. For instance, human BRD4 interacts with acetylated histones and functions as a transcriptional coactivator that stimulates RNA polymerase II-dependent transcription [74]. Although functions of human and yeast homologs have been extensively studied, plant BET proteins have been poorly addressed so far, and only a small number of Arabidopsis homologs has been functionally characterized. In Arabidopsis, 12 BET-encoding genes—named GENERAL TRANSCRIPTION FACTOR GROUP E (GTE) 1–12—have been identified, which are organized in two different phylogenetic clusters (Figure 1): GTE1-7 make their own group, whereas a second group is formed by GTE8-12 [28].

### 4.1. First Cluster of BET Proteins

GTE1 or IMB1 (At2g34900) was the first BET protein characterized in plants [70]. IMB1 localizes in the nucleus and is detected mainly in roots and flowers. However, it is during seed imbibition that *IMB1* transcript demonstrates its highest expression, indicating a specific role in the regulation of seed germination. Although *imb1* adult plants appear normal, *imb1* seedlings exhibit hypersensitivity to ABA [70]. Additionally, *imb1* seeds germinate at significantly lower rates compared to *wt* seeds when exposed to a far-red (FR) pulse; as FR indicates the phyA (phytochrome A)-mediated VLFR (very-low-fluence response), it was concluded that *imb1* plants show deficient phyA-mediated VLFR of germination [70]. Additionally, microarray analysis showed that IMB1 probably functions as a transcription factor, regulating transcript levels of various genes, including genes involved in cell wall metabolism, photosynthesis and lipid metabolism [70].

GTE6 (At3g52280), closely related to GTE1 (Figure 1), is involved in the establishment of leaf shape in Arabidopsis during juvenile-to-adult transition [75]. GTE6 upregulates ASSYMETRIC LEAVES 1 (AS1) during leaf development, a gene involved in the control of cell differentiation in leaves [75,76]. In mature leaves, high GTE6 expression leads to increased histone acetylation of the AS1 promoter and transcribed region, leading to AS1 activation. This results in laminae elongation and the elliptical shape of Arabidopsis mature leaves. In line with this finding, *gte6* mutants show round laminae compared with *wt* plants, which have elliptical laminae. It has been suggested that GTE6 may increase AS1 histone acetylation, acting as a shield that protects acetylated histones by HDACs. Another possibility is that GTE6 acts as an adaptor that facilitates HAT-mediated histone acetylation [75].

Finally, in this cluster of BET proteins, GTE4 (At1g06230) has also been functionally characterized. *gte4* plants demonstrate a delay in germination, are reduced in size at all developmental stages and exhibit smaller and slightly serrated leaves. Roots also appear significantly shorter with quiescent center (QC) cells not functioning properly. All the above are a result of a lower number of cells due to defects in cell proliferation, as GTE4 was shown to be involved in the maintenance of the mitotic cell cycle [77]; *gte4* mutants show a delay in cell cycle activation, as cells exit earlier from the mitotic cell cycle and go from mitosis to endoreduplication [77,78]. Consistent with the defect of *gte4* mutants in root development is the finding that GTE4 is regulated by the auxin-induced CROWN ROOTLESS1 (CRL1), which is required for the initiation of crown root primordia in rice [79]. More recently, a study revealed a role for GTE4 in the regulation of immune response in Arabidopsis. *gte4* mutants appeared more resistant to *Pseudomonas syringe* inoculation, which suggests a negative role of GTE4 in immune responses [80]. GTE4 was shown to mainly act as a transcriptional activator and bind to active genes, including ribosome biogenesis genes; the high expression levels of these genes are maintained during pathogen infection [80]. Furthermore, GTE4 can also act as a transcriptional repressor, as it binds and represses 12-oxophytodienoate reductase OPR3, a protein required for Jasmonic acid (JA) biosynthesis. This leads to the overaccumulation of JA and enhanced JA-responsive gene expression. Consistent to the resistance in *P. syringe*, the elevated JA content in *gte4* mutants is coupled with the downregulation of JA-mediated defense genes and the upregulation of salicylic acid (SA)-mediated defense genes [80].

Although the rest of BET proteins in this group have not been functionally characterized, there are some interesting findings. GTE3 (At1g73150) and GTE5 (At1g17790) have high homology and are the only BET proteins predicted to have a chloroplastic localization (www.uniprot.org, accessed on 11 July 2023). Both interact with the SUMO ligase SAP AND MIZ 1 (SIZ1), which is involved in various stress responses [81], whereas GTE3 was found to interact with acetylhistone H3, and this interaction is weakened by sumoylation [82].

GTE2 (At5g10550) and GTE7 (At5g65630) are closely related and have been shown, together with GTE4, to be involved in Agrobacterium-mediated root transformation in Arabidopsis; however, their exact role remains elusive [83]. GTE2 was found to co-immunoprecipitate with tRNA-specific adenosine deaminase 3 (TAD3) [84], which is involved in stress response, regulation of the cell cycle and DNA metabolism [85]. GTE7 was identified as an interactor of the two-component response regulator ARR6 [86], which is involved in CK signaling, cell wall composition and disease resistance [87]. Furthermore, the tomato ortholog of GTE7, named Viroid-binding protein 1 (VIRP1), was found to localize in the nucleus and specifically interact with pospiviroids, an interaction mediated by an atypical RNA-binding proline-rich domain at the C-terminal of VIRP1 [88]. Viroids are small, circular RNA pathogens that infect several crop plants, causing diseases of economic significance [89,90,91]. VIRP1 was proven substantial for viroid accumulation, as VIRP1-suppressed *Nicotiana benthamiana* plants were found resistant to Potato Spindle Tuber viroid (PSTVd) mechanical inoculation [92]. In later studies, it was demonstrated that VIRP1 mediates—at least partially—the nuclear import of viroids and satellites [93,94]. This observation was further supported by a recent study, where GTE7, the Arabidopsis closest homolog of VIRP1, was shown to also interact with PSTVd RNA [95]. In this work, IMPORTINa-4 (IMPa-4) was found to form a complex with PSTVd and GTE7 to promote nuclear transport [95]. The RNA-binding nature of GTE7/VIRP1 is the only case reported for plant BET proteins and could suggest the existence of endogenous RNAs that interact with BET proteins in plants.

### 4.2. Second Cluster of BET Proteins

The second cluster of the BET family contains proteins mainly involved in ABA response. GTE10 or NPX1 (At5g63320) was found to be induced under stress conditions, such as nutrient deprivation, salt stress and ABA treatment [96]. Furthermore, NPX1 was identified as a nuclear factor that regulates both the expression of ABA signaling genes and ABA biosynthesis since ABA levels were significantly higher in *NPX1*-overexpressing plants compared to *wt* plants [96]. NPX1 did not show any transcriptional activation activity in yeast but was found to act as a transcriptional repressor and can eventually be considered as a negative regulator of ABA signaling [96]. Chen and co-authors showed that histone deacetylase HDA9, in a complex with POWERDRESS (PWR) and WRKY53, catalyzes the removal of histone H3 acetylation and suppresses the expression of NPX1, leading to derepression of downstream target genes and promoting leaf senescence [97]. HIGH EXPRESSION OF OSMOTICALLY RESPONSIVE GENES 15 (HOS15) was also reported to act in the same complex with HDA9-PWR, regulating plant growth and development [98]. HOS15 functions as a positive regulator of leaf senescence in Arabidopsis, and it is suggested that together with the HDA9-PWR complex, it regulates senescence by coordinating histone acetylation status on the promoters of key genes, including NPX1 [98,99]. Notably, NPX1 is the only BET protein that is encountered as an interactor of the SWR1 chromatin-remodeling complex, as discussed in Section 3.2 [67].

GTE9 (At5g14270) and GTE11 (At3g01770) were shown to interact in vitro with the N-terminal region of BT2, a BTB/POZ domain-containing protein [71]. BT2 is involved in responses to sugar and ABA in Arabidopsis, and both *gte9* and *gte11* mutants mimic *bt2* responses for seed dormancy and inhibition of seedling growth under glucose and ABA treatment [100]. Furthermore, *gte9* and *gte11* mutant seeds were more sensitive to ABA inhibition of germination compared to *wt* seeds [71]. In contrast, overexpression of *GTE9* and *GTE11* resulted in resistance to glucose and ABA-mediated inhibition of germination, in a way similar to *BT2* overexpression [71]. Therefore, it was demonstrated that GTE9 and GTE11 are negative regulators of sugar and ABA signaling and are required for BT2-mediated resistance to glucose and ABA [71]. In a more recent work, BT2 was shown to assemble into a functional complex involving GTE9, GTE11 and CULLIN3, driving a 35S enhancer function in Arabidopsis [101].

## 5. Bromodomain Proteins with Histone Acetyltransferase Activity

HATs and HDACs are the two groups of enzymes that work together as the “writers” and “erasers” of histone acetylation, respectively; HATs add acetyl groups to lysine residues on histones, whereas HDACs remove them. The loosening of chromatin structure brought on by HATs’ addition of acetyl groups to histones facilitates gene activation [102]. According to their localization and substrate preference, HATs are divided into A-type enzymes (HAT-A), which are found in the nucleus and acetylate the nucleosome core histones, and B-type enzymes (HAT-B), which are found in the cytoplasm and target free histones [102]. The HAT-A class is further classified into four families: (1) GENERAL CONTROL NON-REPRESSIBLE 5 (GCN5)-related N-terminal acetyltransferase (GNAT) family, (2) MYST (MOZ, Ybf2/Sas3, Sas2 and Tip60) family, (3) p300/CREB binding protein (CBP) family and (4) TATA-binding protein-associated factor (TAFII250) (TAF) family [103].

A comparison of Arabidopsis HAT and HDAC genes with those in other eukaryotic genomes revealed that both groups of genes have expanded and diversified significantly in multicellular eukaryotes since the divergence of plants, animals and fungi [104]. The Arabidopsis genome encodes four GNAT members, named HISTONE ACETYLTRANSFERASE OF THE GNAT FAMILY (HAG or GCN5) 1, 2 and 3 and MEIOTIC CONTROL OF CROSSOVERS 1 (MCC1); two MYST family genes, named HISTONE ACETYLTRANSFERASE OF THE MYST FAMILY 1 and 2 (or HAM1 and HAM2); five CBP family genes, named HISTONE ACETYLTRANSFERASE OF THE CBP FAMILY 1, 2, 4, 5 and 12 (or HAC1, HAC2, HAC4, HAC5 and HAC12); and two TAFII250 family genes, named HISTONE ACETYLTRANSFERASE OF THE TAFII250 FAMILY 1 (HAF1 or TAF1) and 2 (HAF2 or TAF1b) [104,105]. Of these, three genes encode BRDs: HAG1/GCN5, HAF1/TAF1 and HAF2/TAF1b.

### 5.1. GNAT Family: HAG1 Histone Acetyltransferase

The most well-studied HAT in eukaryotes is HAG1 or GCN5 (At3g54610) [106]. The first evidence linking histone acetylation to the regulation of gene transcription is found in a study on the HAT activity of *Tetrahymena thermophila* HAG1 [102]. HAG1 is a key catalytic component of acetyltransferase complexes [107]. In particular, HAG1 is an important member of the SAGA (Spt-Ada-Gcn5-acetyl-transferase) complex, a multi-subunit complex with HAT activity, highly conserved among eukaryotes. The SAGA complex was found to stimulate the transcription of stress-related genes in budding yeast, some of which require the BRD of the HAG1 subunit for full activation [108]. HAG1 BRD specifically regulates lysine acetylation on histone H3, and the presence of both HAT and BRD domains makes HAG1 a “reader” and “writer” of epigenetic marks [109]. The BRD of HAG1 has various different roles in the formation of nucleosomal acetylation in the SAGA complex, and it is necessary for the acetylation in nucleosomes that start out non-acetylated [110].

In plants, HAG1 is involved in a wide range of critical biological processes, including growth and development, cell differentiation, secondary metabolism and stress responses [111]. Arabidopsis HAG1 actively regulates plant root stem cell maintenance via the control of the stem cell transcription factors PLETHORA1 (PLT1) and PLETHORA2 (PLT2) gene expression [112]. HAG1 has also been proved crucial for cell division and proliferation in rice, where it is highly expressed in the root meristem [113]. The rice ALTERATION/DEFICIENCY IN ACTIVATION 2 (ADA2) and HAG1 regulate a set of root-specific genes that are essential for root growth, including those involved in energy metabolism, cell wall synthesis and hormone responses [113]. In Arabidopsis, HAG1 and ADA2b are positive regulators of cytokinin signaling during root growth by histone acetylation of cytokinin-related genes [114]. Further to that, the acquisition of callus pluripotency and subsequent shoot regeneration depend on HAG1, which acts as an epigenetic switch for cell proliferation [115]. HAG1-mediated histone acetylation assists the activation of transcription factors encoded by the root-meristem genes, which function as potency factors, giving callus cells pluripotency and allowing successful shoot regeneration [115]. HAG1 has also been shown to directly affect light-responsive promoters, regulating photomorphogenesis and light-regulated gene expression to control plant development [116]. Developmental defects such as dwarfism, leaf folding and serration, loss of apical dominance and flowering abnormalities can all result from HAG1 mutations. These changes ultimately affect the overall vegetative growth [117].

Aiming for a smooth transition from the juvenile to adult phase HAG1 and the ADA2b factor of the SAGA complex regulate the SQUAMOSA PROMOTER BINDING PROTEIN-LIKE (SPL) plant genes [118]. *SPL* gene expression must increase while *miR156* (a microRNA targeted by SPLs) expression decreases, so that Arabidopsis plants can progress through the three distinct stages of growth and development: juvenile to adult, primary inflorescence and floral meristem identity [119]. Throughout this process, HAG1 and ADA2b maintain high histone acetylation levels of SPLs, which results in the necessary increase of SPL expression. By decreasing the expression of miR156-targeted SPL genes as plants age, plants achieve the *miR156*-mediated regulation that defines an age-dependent inflorescence pathway. In addition, *miR156*-independent activation of SPLs by environmental signals that accelerate phase transitions also require HAG1-mediated histone acetylation [118].

Regarding its function in stress responses, HAG1 significantly affects the resistance to abiotic stresses by maintaining the homeostasis of key metabolites through the regulation of stress-related gene expression [120]. In Arabidopsis, its function is essential for the transcriptional activation of heat stress and salt stress-related genes [121,122]. Upon low temperature conditions, HAG1 along with ADA2b also regulate the expression of genes that are related to cold stress responses [117].

### 5.2. TAFII250 Family: HAF1 and HAF2 Histone Acetyltransferases

Arabidopsis encodes two TAFII250 isoforms: HAF1/TAF1 (At1g32750) and HAF2/TAF1b (At3g19040), hereafter referred to as HAF1 and HAF2, respectively. Together with RNA polymerase II and a subset of other essential transcription factors, TAFII250 family proteins form the pre-initiation complex upon transcription initiation [123]. HATs from the TAFII250 family have been shown to contain one HAT domain, two kinase domains and two BRDs with affinity for highly acetylated H4 histone [124]. A characteristic TAF N-terminal domain is found in Arabidopsis, rice and Drosophila HAF1 but is mostly absent in the HAF2 isoform [125]. This domain was shown to be important for the interaction with TATA-binding protein (TBP) in Drosophila (Figure 1) [125].

In response to plant genome damage, HAF1 C-terminal BRD plays a significant role. Although C-terminal BRD-deficient Arabidopsis *haf1* mutants do not exhibit developmental abnormalities [126], they are more susceptible to DNA damage hypersensitivity, which may be caused by transcriptional dysregulation of the DNA damage response [123]. HAF1 is also considered to have a significant role in UV-B signaling and response. *haf1* mutants showed less inhibition of root and leaf growth after UV-B treatment experiments [127]. In this context, HAF1 was shown to specifically affect cell endoreduplication, possibly through interactions with specific transcription factors known as E2 Promoter Binding Factors (E2F). Under normal growth conditions, HAF1-deficient plants presented decreased endoreduplication and cell area, but after UV-B exposure, ploidy levels were restored and cell area increased [127].

HAF2 has certain roles during plant development by mediating light responses. Since HAF2 is required for leaf greening and light-induced transcription, its absence causes a variety of developmental defects. It particularly functions as a coactivator of light-induced gene transcription by histone acetylation and integration of light signals [126]. Importantly, HAG1 and HAF2 both affect histone acetylation on light-inducible promoters and show cumulative effects on plant growth and light-responsive gene expression, suggesting that they functionally interact as light-responsive coactivators [116]. HAF2 is activated at midday and is a key player in the circadian clock regulation; an element called CIRCADIAN CLOCK-ASSOCIATED 1 (CCA1) regulates the timing of the HAF2 expression [128]. In order to achieve precise circadian oscillation, HAF2 promotes H3 acetylation deposition at the promoters of two core circadian clock activity-related loci, named PSEUDO-RESPONSE REGULATOR 5 (PRR5) and LUX ARRHYTHMO (LUX). This results in enhanced gene activation, indicating that modifications to chromatin structure are responsible for the temporal coordination of circadian components [128].

## 6. Other Bromodomain Proteins

In this section, we aimed to include two additional BRD-proteins, which cannot be incorporated to any of the above functional groups. The first one is named Bromodomain-containing protein with ATPase domain 1 (BRAT1, At1g05910) and contains both a BRD and an ATPase domain [129]. The simultaneous presence of both domains in a single module remains rather atypical, and only few proteins have been described so far. For instance, human ATAD2 (ATPase family AAA+ domain-containing protein 2) has been described as a transcriptional coactivator for chromatin modifications [130] and is found upregulated in a wide range of cancers [131]. In yeast, Yta7 (yeast Tat-binding analog 7) has been involved in transcriptional induction of histone genes and early meiotic genes, as well as nucleosome disassembly [132,133]. Moreover, in *Caenorhabditis elegans*, LEX-1 has been proved to influence gene expression of repetitive sequences [134]. BRAT1 was identified in Arabidopsis as an anti-silencing factor, acting together with its cofactor BRAT1 partner 1 (BRP1), which also carries an ATPase domain. The BRAT1-BRP1 complex binds to acetylated histone H4 through BRAT1 BRD and further promotes histone acetylation and thus transcriptional activation, preventing transcriptional silencing at methylated DNA loci [129].

The second protein was identified in *N. benthamiana*, named DNA-binding bromodomain-containing protein (DBCP, Niben101Scf17137g00006.1). DBCP carries a SANT (Swi3, Ada2, N-Cor, a TFIIIB domain-type helix-turn-helix), a coiled-coil and a BRD domain [135]. In Arabidopsis, there are four BRD-proteins with a SANT and a coiled-coil domain, with two of them carrying an additional C-terminal lysine-rich domain (Figure 1). SANT domains, present in numerous proteins of various organisms, are protein–protein interaction modules that link histone tail-binding to chromatin remodeling [136,137]. The simultaneous presence of a SANT and a BRD in the same module is an uncommon feature and, to our knowledge, has not been described in animals or yeast but is relatively common in plant species, including tomato, potato and rice [135]. Although the physiological roles of this group of proteins remain elusive, DBCP was identified as a repressor of Potato virus X (PVX) immune response. DBCP was found to localize close to histones in the nucleus and nucleolus, interacting directly with Rx1 through its BRD [135]. Rx1 is an NLR (Nucleotide-binding Leucine Rich repeat-type immune receptor) protein that confers resistance to potato virus X (PVX) after recognition of its coat protein [138]. Rx1 binds and distorts double-stranded DNA upon immune activation and negatively regulates DNA-binding of transcriptions factors (e.g., NbGolden2-like/Glk1) [138]. DBCP was shown to act as a negative regulator of Rx1-mediated immunity through alteration of Glk1 DNA binding through its BRD domain. This finding provides for the first time a putative link between chromatin state and NLR activity [135].

## 7. Discussion

Plants encode a significant number of BRD-proteins, yet many of them are not characterized. We divided the studied plant BRD-proteins in functional groups: (a) BRD-proteins of chromatin-remodeling complexes; (b) BET family transcriptional regulators; (c) BRD-proteins with histone acetyltransferase activity; (d) other BRD-proteins (Figure 2). With this review, our aim is to concentrate the current literature on plant BRD-proteins and highlight similarities and unique features compared to animal or yeast counterparts, as well as key questions for future research.

The role of the bromodomain is mainly the recognition of acetylated lysines in histones [15], and plant BRD-proteins, similarly to their animal homologs, participate in various processes. BRDs in plants, similarly to animals or yeast, may exist alone or coexist with a variety of other domains in the same protein [29]. A characteristic difference between plant BRD-proteins when compared to human or yeast BRDs is the presence of a single bromodomain instead of two. Thus, it is expected that multiple plant BRD-proteins function as homologs of polybromoproteins found in animals [20]. Interestingly, SANT domains are common in animal proteins [136] but have not been encountered to coexist with bromodomains in the same module, whereas in Arabidopsis, four such proteins can be found [28,29], with yet unexplored functions. Given that the SANT domain can recognize and bind to specific histone modifications and is related to chromatin remodeling [136], it would be tempting to speculate that in some cases, it could cooperate with a single BRD. Another finding that supports the above hypothesis is that BRD1, 2 and 13 are additional members of the SWI/SNF complex in Arabidopsis, cooperating with BRM [59]. Finally, one more such example can be given by the fact that the histone acetyltransferases HAG1 and HAF2 functionally interact to modify histone acetylation on light-inducible promoters [116]. It has been suggested that histone acetylation by either of these two HATs could induce binding of the other to chromatin, thus enhancing histone acetylation on the promoters of target genes [116]. This speculation further supports the theory of multiple plant BRD-proteins cooperating due to the absence of multiple BRDs in a single protein module.

An important field for future research is of course the elucidation of the endogenous roles of yet unstudied plant BRD proteins. For example, it is predicted that GTE3 and GTE5 are likely chloroplastic; however, they may also have at least a partial presence in the nucleus, given that they interact with the nuclear sumo ligase SIZ1 [82]. The intriguing possibility that these two proteins function in both the nucleus and chloroplast renders them compelling subjects for further study. Furthermore, GTE2 and GTE7, two closely related BET proteins, have yet to be investigated. Notably, the binding of GTE7 to PSTVd RNA implies the possibility of other endogenous RNA molecules associated with GTE7 through the same proline-rich domain responsible for RNA-binding—a domain that is also present in GTE2. In support of this hypothesis, BRD4—the most well-studied animal BET—was found to bind with several long non-coding RNAs, which affect BRD4 transcriptional gene regulation [139]. It is worth mentioning that although Arabidopsis encodes relatively fewer BRD-proteins (29) compared to humans (46), 12 BETs have been identified, a number surprisingly high compared to the 4 human BETs. This could suggest a diversification of BET protein functions in plants that still awaits to be explored.

Outside the BET family, two BRD-proteins with WD40-repeats have been identified in Arabidopsis, with their function remaining obscure (Figure 1) [28,29]. An example of such a protein in humans is BRWD3, containing WD40 tandem repeats and a C-terminal BRD. In human cells, BRWD3 plays a role in the regulation of cell morphology and cytoskeletal organization, affecting cell shape [140]. It would be intriguing to explore the function and interacting partners of WD40/BRD proteins in plants.

BRD-proteins with a SANT N-terminal domain are present in plants, as discussed above, and have not been studied either. This group of proteins might unveil unique functions of plant BRD-proteins, as the coexistence of SANT and BRD domains is a feature that is not present in human BRDs.

Apart from the yet uncharacterized plant BRD-proteins, other important questions are: How do BRD-proteins cooperate to succumb the presence of a single BRD instead of two? What are the common functions between plant and animal or yeast BRDs, and which features are unique?

Of course, there are a number of limitations regarding the research on the field of plant BRD-proteins. First of all, in other model plants beside Arabidopsis, the genome annotation is still not complete, which makes plant research more difficult in general. Furthermore, the genetic complexity of plant genomes, with many of them being polyploidy, makes it even more difficult to decipher the complex networks underneath. More specifically regarding epigenetic regulation, it is challenging to study and manipulate epigenetic modifications in plants, as they are greatly influenced by environmental factors.

Overall, plants harbor a wide array of BRD-proteins with diverse functions that hold great potential for exploration. Intriguingly, these proteins exhibit several distinct features that have not been encountered in other organisms, implying the presence of unique mechanisms underlying the functions of plant BRD-proteins. Given that several plant BRD-proteins are emerging as regulators of stress responses or host factors linked to pathogen infections, plant BRD-genes could be considered promising as potential breeding targets for resistance to biotic or abiotic stress. To fully comprehend the intricate network established by their bromodomains, further investigations are required, not only in Arabidopsis but also in other plant species. Such studies will shed light on the complex interplay and functionalities orchestrated by these remarkable proteins in plants.

## Data Availability

The publications used for this research are all properly cited. No other data were used in this work.
